# C:N:P stoichiometry in plant, soil and microbe in *Sophora moorcroftiana* shrubs across three sandy dune types in the middle reaches of the Yarlung Zangbo River

**DOI:** 10.3389/fpls.2022.1060686

**Published:** 2023-01-11

**Authors:** Ruizhen Dong, Shihai Yang, Xiaoli Wang, Lele Xie, Yushou Ma, Yanlong Wang, Litian Zhang, Min Zhang, Jinping Qin

**Affiliations:** ^1^ Qinghai Provincial Key Laboratory of Adaptive Management on Alpine Grassland, Qinghai Academy of Animal and Veterinary Science, Qinghai University, Qinghai Province, Xining, Qinghai, China; ^2^ Tibet Yunwang Industrial Co., Ltd, R & D Department, Shigatse, Tibet, China

**Keywords:** *Sophora moorcroftiana* population, sandy dune type, C:N:P ecological stoichiometry, nutrient limitation, plant-soil-microbe interaction

## Abstract

The alpine sandy dune ecosystem is highly vulnerable to global climate change. Ecological stoichiometry in plants and soils plays a crucial role in biogeochemical cycles, energy flow and functioning in ecosystems. The alpine sandy dune ecosystem is highly vulnerable to global climate change. However, the stoichiometric changes and correlations of plants and soils among different types of sandy dunes have not been fully explored. Three sandy dune types (moving dune, MD; semifixed dune, SFD; and fixed dune, FD) of the *Sophora moorcroftiana* shrub in the middle reaches of the Yarlung Zangbo River were used as the subjects in the current study. Plant community characteristics, soil physicochemical properties, carbon (C), nitrogen (N), and phosphorus (P) contents of leaves, understorey herbs, litter, and soil microbes were evaluated to explore the C:N:P stoichiometry and its driving factors. Sandy dune type significant affected on the C:N:P stoichiometry in plants and soils. High soil N:P ratio was observed in FD and high plant C:P and N:P ratios in SFD and MD. The C:N ratio decreased with sand dune stabilization compared with other stoichiometric ratios of soil resources. Leaf C:P and N:P ratios in *S. moorcroftiana* were higher than those in the understorey herb biomass, because of the low P concentrations in leaves. C, N and P contents and stoichiometry of leaves, understorey herbs, litter and microbe were significantly correlated with the soil C, N and P contents and stoichiometry, with a higher correlation for soil N:P ratio. P was the mainly limiting factor for the growth of *S. moorcroftiana* population in the study area and its demand became increasingly critical with the increase in shrub age. The variation in the C:N:P stoichiometry in plants and soils was mainly modulated by the soil physicochemical properties, mainly for soil moisture, pH, available P and dissolved organic C. These findings provide key information on the nutrient stoichiometry patterns, element distribution and utilization strategies of C, N and P and as well as scrubland restoration and management in alpine valley sand ecosystems.

## 1 Introduction

Carbon (C), nitrogen (N), and phosphorus (P) are key elements in living organisms (Chen and Chen, 2021). Carbon provides the structural basis of plants and accounts for relatively 50% of dry plant biomass (Schade et al., 2003; Yang et al., 2018). Nitrogen is an essential component of proteins and plays an important role in plant production, photosynthesis and litter decomposition (Daufresne, 2004; Chen et al., 2016b; Yang et al., 2018). Phosphorus is the limiting element responsible for the structure of cells and the composition of DNA and RNA, and P promotes C/N transpiration and assimilation (Tilman, 1996; Xiao et al., 2009; Pii et al., 2015). Nutrient sources have differences in the elemental composition; however, living organisms maintain these elements around specific ratios for their growth and reproduction in a process known as homeostasis (Sterner and Elser, 2002).

C:N:P ecological stoichiometry comprises on the interaction and balance of chemical elements in ecological processes and is used to study feedback and relationships between above- and belowground components of ecosystems (Mooshammer et al., 2014; Ren et al., 2016; Zhang et al., 2019). Two major methods are used for analysis of C:N:P stoichiometry (Cross et al., 2005). The first method relies on the principle that release of major nutrients (C, N, and P) occurs in a predictable manner, depending on the limitation of the C:N:P ratio (Sterner and Elser, 2002; Yang et al., 2018). Leaf N:P ratio (mass ratio) is utilized as a key indicator for assessing N or P limitation (Güsewell, 2004; Cleveland and Liptzin, 2007). The second method is based on the theory that natural resources are in stoichiometric homeostasis (Sterner and Elser, 2002; Yang et al., 2018). At the organismal level, life stages characterized by high growth rates are associated with high RNA contents (C:N:P ratios ranging from 18:6:1 to 21:7:1), and thus organisms can alter their overall stoichiometry (McGroddy et al., 2004). Several studies have demonstrated that the C:N:P ratios in plants, soils and soil microorganisms affect primary production and nutrient cycling as well as indicate the nutrient limits in plants in terrestrial ecosystems (Sterner and Elser, 2002; Manzoni et al., 2008; Huang et al., 2013). The relative supplies of nutrients (soil N and P) are key modulators of C:N:P ratios in plants (Plum et al., 2015). Plant C:N:P ratios can be transformed through litter decomposition into soil inputs and microbes (Zechmeister-Boltenstern et al., 2015). Researchers have demonstrated that more diverse plant assemblages can increase the C:nutrient (N or P) ratios of plants and associated litter (Mooshammer et al., 2014). The high plant C:nutrient (N or P) ratios in turn increase the C:N and C:P ratios in soil microbial biomass because microorganisms adjust their biomass C:N:P ratios to match the litter substrate (Zechmeister-Boltenstern et al., 2015). Stoichiometric imbalances in the plant–soil–microbe system have not been fully elucidated (Mooshammer et al., 2014; Zhang et al., 2019). Studies should be conducted to explore how plant diversity, successional stage, and environmental factors affect redistribution of nutrients (C, N, and P) between plants and soils (Zhang et al., 2019), and how relationships between above- and belowground elemental stoichiometry modulate terrestrial ecosystem processes (Li et al., 2016; Liu et al., 2020). Evaluation of the C:N:P stoichiometry of plants, soils, and soil microbial biomass will provide insights into element cycling and promote ecosystem sustainability (Chen and Chen, 2021).

In semiarid ecosystems, nurse shrubs facilitate the establishment of other plant species under their canopies and induce changes in the understory plant communities as they grow (Hortal et al., 2013). Studies should be conducted to predict the feedback effects of shrub/understorey species interactions (Bai et al., 2018). Understorey herbaceous plants overall negative, neutral, and positive effects on shrub growth and reproductive output, exhibiting antagonistic, commensalistic, and facultative mutualistic relationships (Bai et al., 2019). Moreover, understorey herbaceous plants are crucial in increasing soil nutrient contents and promoting nutrient cycling among plants and soil systems (Zhang et al., 2019). Nurse shrubs mitigate the responses of understorey herbaceous species to N enrichment and increased precipitation (Bai et al., 2019). Therefore, it is imperative to explore the biochemical cycle of ecosystems using stoichiometry theory to evaluate the internal relationship between understorey species and shrubs and their relationship with soil elements.

The Tibetan Plateau is characterized by excessive land desertification and is one of the most ecologically vulnerable regions (Shen et al., 2012). Degradation of alpine grasslands causes a reduction in ecosystem services, such as the loss of biodiversity, water erosion and reduced productivity (Du and Gao, 2021). Shrub species in dune habitats are the key elements in rehabilitating a degraded ecosystem (Liao et al., 2021). This is because shrubs have specific and significant “morphological, physiological or behaviour” effects in these extreme and harsh environmental conditions (Yu and Steinberger, 2012). These adaptations markedly affect the substrate stability and sand movement as well as the basal soil organic matter (SOM), nutrient contents, salinity and other conditions, and affect the activity, quantity and diversity of soil microorganisms (Wang et al., 2019). *Sophora moorcroftiana* (Benth.) Baker, a perennial leguminous shrub, is an endemic species in Tibet region. The species is limited to the hillsides, sandy gravel floodplains, sandy areas and alluvial fans of the wide valley in the middle reaches of the Yarlung Zangbo River (Cheng et al., 2017). It is one of the most important sand-fixing plant species characterized by high tolerances to drought, cold, barren land and sand burial, as well as acts as a key species for vegetation restoration and reconstruction in this region (Zhao et al., 2007; Guo et al., 2014). The species plays an essential role in water and soil conservation, fixation of sand dunes and prevention of shifting sands in the middle reaches of the Yarlung Zangbo River (Guo et al., 2009). However, the ecological effects of the interrelationships among this shrub, understorey species, litter, soil and microbes in the different sandy dune types have not been fully evaluated from the perspective of C:N:P stoichiometry.

In this study, we evaluated the dynamics of C, N, and P contents and stoichiometry in plants (including green leaves, understorey species, and litter), soils and microbial biomass from three different sandy dune types (mobile, semifixed, and fixed dunes) in an *S. moorcroftiana* population. We hypothesized that the C:N:P stoichiometry in the shrub, understorey species, litter, soils and microbes are affected by the sandy dune type, and the C, N, and P concentrations and their ratios in the soils, plants and microbes are highly coupled because they coevolved. In addition, we also presumed that soil properties are a key driver of C:N:P stoichiometry in plant-soil-microbe systems. Therefore, the objectives of the present study were to (1) evaluate the effect of sandy dune type on C:N:P stoichiometry in plants, soils, and microbes and determine the element limiting the growth of *S. moorcroftiana* shrubs and understorey plants; (2) explore the relationships of C:N:P stoichiometry between plants, soils, and microbes; and (3) determine the driving factors of the C:N:P stoichiometry.

## 2 Materials and methods

### 2.1 Study area

This study was conducted at the southern Tibetan Plateau in the middle reaches of the Yarlung Zangbo River (28°59′–29°26′ N, 88°04′–93°18′ E) (**Table 1**). The terrain of the catchment area is higher in the northwest and lower in the southeast, with an altitude ranging from 2,900–4,550 m. This terrain provides significant negative topographic conditions for the accumulation of eolian sediments, and eolian deposits (such as eolian sand and sandy loess), which are widely distributed in the basin. The river basin is characterized by strong winds during winter and spring. The average wind velocity is extremely high at 32.5 m/s, the monthly maximum wind velocity is 4.7 m/s, and the number of windy days is about 172 days per year (Shen et al., 2012). The region has a temperate semihumid and semiarid plateau monsoon climate. The average annual temperature is approximately 6–8°C, with summer temperatures above 15°C and winter temperatures below -2°C. The average annual precipitation is 300–600 mm, and the precipitation around the basin is unevenly distributed throughout the year, mainly occurring from June to September, which accounts for 86.6% of the annual precipitation. The soil type in this area is primarily mountainous shrub steppe soil and alpine meadow soil, and some areas have alpine cold desert soil, subalpine meadow soil, and subalpine steppe soil. The area has low and sparse vegetation, with grassland as the main vegetation type. The main vegetation in the middle reaches of the Yarlung Zangbo River are shrubs dominated by *S. moorcroftiana* (**Table 1**). *Pennisetum centrasiaticum*, an important companion species, is distributed in all three sandy dune types, but *Agriophyllum squarrosum* is only distributed in the semifixed dune type and moving dune types. *P. centrasiaticum* and *Artemisia wellbyi* were the main companion species in the semifixed dune type and moving dune types. Several new species, such as *Thermopsis fabacea*, *Setaria viridis*, *Erodium stephanianum*, and *Achnatherum splendens*, were observed in the fixed dune type, with *P. centrasiaticum*, *Arisaema flavum*, *A. wellbyi*, *Orinus thoroldii*, and *Artemisia stechmanniana* as the main companion species.

**Table 1:** Main geographic characteristics and floristic compositions of the *S.moorcroftiana* shrubs sampling sites among the middle reaches of the Yarlung Zangbo River basin.

Sites	Longitude (E)	Latitude (N)	Elevation (m)	Main companion species	Minor species
FD1	88°31’54.74”	29°11’00.39”	3957	*Pennisetum centrasiaticum*	*Poaannua*, *Astragalus membranaceus*
FD2	88°38’04.54”	29°09’11.07”	3964	–	–
FD3	89°20’30.35”	29°05’17.94”	3886	*Pennisetum centrasiaticum*	*Astragalus membranaceus*
FD4	87°27’38.31”	29°06’09.21”	3998	*Pennisetum centrasiaticum*	*Thermopsis fabacea*
FD5	88°09’27.16”	29°21’14.97”	3886	*Pennisetum centrasiaticum*	*Astragalus membranaceus*, *Artemisia scoparia*
FD6	89°23’52.30”	29°22’09.29”	3806	*Arisaema flavum*	*Pennisetum centrasiaticum*, *Dysphania schraderiana*, *Artemisia scoparia*, *Orinus thoroldii*
FD7	89°35’44.24”	28°55’46.39”	4019	*Pennisetum centrasiaticum*	*Artemisia stechmanniana*, *Artemisia wellbyi*, *Astragalus membranaceus*
FD8	89°45’24.52”	29°18’15.28”	3745	–	*Pennisetum centrasiaticum*, *Arisaema flavum*, *Artemisia scoparia*, *Setaria viridis*
FD9	90°10’39.67”	29°26’16.11”	3785	*Pennisetum centrasiaticum*	*Artemisia wellbyi*
FD10	90°50’54.31”	29°16’51.66”	3543	*Pennisetum centrasiaticum*	*Astragalus membranaceus*, *Tribulus terrestris*, *Erodium stephanianum*
FD11	90°54’15.75”	29°20’00.87”	3539	*Pennisetum centrasiaticum*	*Artemisia wellbyi*, *Heteropappus gouldii*, *Oxytropis sericopetala*
FD12	91°21’19.16”	29°18’24.35”	3534	*Artemisia wellbyi*	*Pennisetum centrasiaticum*, *Ceratostigma minus*, *Achnatherum splendens*
FD13	91°53’45.30”	29°16’57.26”	3535	*Pennisetum centrasiaticum*, *Orinus thoroldii*	*Artemisia stechmanniana*, *Artemisia wellbyi*, *Artemisia demissa*, *Achnatherum splendens*
FD14	92°43’02.36”	29°06’11.87”	3126	*Artemisia stechmanniana*	*Artemisia wellbyi*, *Arisaema flavum*
FD15	93°18’21.68”	28°59’37.45”	3032	*Artemisia wellbyi*, *Artemisia stechmanniana*	*Pennisetum centrasiaticum*, *Poaannua*, *Astragalus membranaceus*
SFD1	91°05’06.60”	29°20’15.46”	3543	–	*Oxytropis sericopetala*, *Artemisia wellbyi*
SFD2	91°09’09.82”	29°19’35.75”	3543	*Orinus thoroldii*	*Pennisetum centrasiaticum*, *Astragalus membranaceus*, *Oxytropis sericopetala*, *Agriophyllum squarrosum*, *Trikeraia hookeri*
SFD3	91°30’03.92”	29°19’58.66”	3530	*Pennisetum centrasiaticum*, *Artemisia wellbyi*	*Oxytropis sericopetala*, *Astragalus membranaceus*
SFD4	91°31’42.81”	29°15’19.90”	3523	*Pennisetum centrasiaticum*	*Dysphania schraderiana*, *Artemisia wellbyi*, *Poaannua*
MD1	88°04’20.50”	29°21’53.67”	3884	–	*Oxytropis sericopetala*, *Astragalus membranaceus*, *Artemisia scoparia*, *Pennisetum centrasiaticum*
MD2	89°30’27.56”	29°21’40.85”	3750	*Pennisetum centrasiaticum*	*Oxytropis sericopetala*
MD3	91°21’05.25”	29°18’45.76”	3553	*Pennisetum centrasiaticum*	*Agriophyllum squarrosum*, *Artemisia wellbyi*, *Oxytropis sericopetala*
MD4	91°31’42.81”	29°15’19.90”	3523	*Artemisia wellbyi*	*Oxytropis sericopetala*, *Tribulus terrestris*, *Pennisetum centrasiaticum*, *Dysphania schraderiana*
MD5	91°35’20.60”	29°15’14.44”	3548	*Agriophyllum squarrosum*	*Pennisetum centrasiaticum*, *Saussurea arenaria*, *Dysphania schraderiana*

FD, fixed dune; SFD, semifixed dune; MD, moving dune.

### 2.2 Experimental design

Field plant collection and soil sampling were conducted in August 2020. We selected three sandy dune types, including fixed dune (FD), semifixed dune (SFD), and moving dune (MD), in which *S. moorcroftiana* was the dominant species, according to the vegetation coverage and degree of soil surface fixation in the Yarlung Zangbo River basin (Shen et al., 2012; **Table S1**). Three independent replicate plots of 10 m × 10 m were randomly set up at each site, with five subplots (1 m × 1 m) at the centre and opposite corners of each plot for the subsequent plant sample collection and soil sampling. All the selected sites had similar topographies (flat sandy land) to ensure comparability of the plant and soil samples. Basic information for each sandy dune type of *S*. *moorcroftiana* shrub is presented in **Table 1**.

### 2.3 Soil and plant sampling

Nine soil cores (5 cm diameter) were obtained from each plot (10 m × 10 m) at a depth of 20 cm along an “S” shape under the canopy of the shrub and then thoroughly mixed and pooled to form one soil sample. A total of 72 soil samples (twenty-four replicate sites × three replicate plots) were collected in this study. In addition, three cutting ring soil samples were collected from a 0–20 cm depths in each plot to determine the soil bulk density (BD). Each soil sample was sieved through a 2 mm mesh, and visible gravel and plant materials were removed and then divided into two parts: One portion of the fresh soil from the field was stored at 4°C for microbial biomass (carbon, nitrogen, and phosphorus), inorganic N ( NH_4_^+^−N and NO_3_^*－*^-N ) and available phosphorus (AP) analyses, and the other portion was air-dried and stored at room temperature for subsequent analysis of soil physicochemical properties.

For *in situ* analysis of plants, five subplots (1 m × 1 m) were used to determine the name, height, coverage, and number of each species and to collect litter from the plants. We randomly chose 8–10 well-grown *S*. *moorcroftiana* shrubs in each plot after the basic vegetation survey. Samples of fully expanded leaves were collected, and the diameter at breast height (DBH), height (SH), and shrub crown size (SCS) were determined to characterize the shrub age class as reported previously (Pugnaire et al., 1996; Hortal et al., 2013). All the plant samples were oven-dried at 65°C to a constant mass. The plant samples were analyzed as previously described (Higgins et al., 2012).

### 2.4 Analysis of plant and soil physicochemical properties

Soil samples were collected in a steel ring (Volume 100 cm^3^) for soil BD determination. The soil samples were then oven dried at 105°C (± 2°C) for 24 h, and the dry mass was weighed. Soil pH was determined in a 1:5 (soil:water) ratio after shaking the samples for 30 min at room temperature. The soil moisture (SM) content was determined by oven drying the soil samples at 105°C to constant mass. Organic carbon concentrations of soils and plant samples were assayed using K_2_Cr_2_O_7_ oxidation and FeSO_4_ titration methods (Huang et al., 2014). Total nitrogen (TN) was determined using Cleverchem 200+ (Chilehaus A, Fischertw iete2-20095 Hamburg, Germany) after digestion with H_2_SO_4_-H_2_O_2_. Total phosphorus (TP) concentration was determined using the molybdenum antimony colorimetric method following digestion with H_2_SO_4_-HClO_4_. Soil dissolved organic carbon (DOC) was extracted with 0.5 M K_2_SO_4_ and analysed using vario TOC cube (Elementar, Germany). The soil inorganic N (IN) concentration was extracted with 1 M KCl for 30 min on a shaker at room temperature, and the filtrates were analysed using Cleverchem 200+ (Chilehaus A, Fischertw iete2-20095 Hamburg, Germany). Available P (AP) concentration was determined using the molybdenum blue method (Yang and Liu, 2019).

### 2.5 Determination of soil microbial biomass

Soil microbial biomass carbon (MBC), nitrogen (MBN) and phosphorus (MBP) were determined by the chloroform fumigation extraction method (Brookes et al., 1985; Vance et al., 1987; Wu et al., 2000). Three 30 g soil subsamples for each soil sample were fumigated with chloroform for 24 h at 25°C under dark conditions. Three replicates of the same amount of soil without fumigation were used to extract MBC and MBN using 0.5 M K_2_SO_4_ (of 1:2 soil to acid ratio) for 30 min on a shaker. The extracts were analysed for total organic C and total N using a TOC analyser (vario TOC, Elementar, Germany). MBP was extracted with 0.5 M NaHCO_3_ (pH = 8.5, at a soil solution ratio of 1:20) for 30 min on a shaker. The NaHCO_3_ filtrates (25 ml) were digested with 1 ml 33% H_2_SO_4_ and 4 ml 5% potassium persulfate at 120°C for 30 min (high temperature, high pressure, humid-heat treatment) then the extractions were analysed colorimetrically using a spectrophotometer (Analytikjena, SPECORD 210, Germany) at 880 mm. MBC, MBN and MBP were presented as the difference between levels of fumigated and unfumigated soil samples and expressed as extraction efficiency (conversion coefficient *k*) (*k*_C_ = 0.45 for MBC, *k*_EN_ = 0.45 for MBN, *k*_P_ = 0.40 for MBP) (Brookes et al., 1985; Vance et al., 1987; Joergensen and Mueller, 1996).

### 2.6 Statistical analysis

Statistical analyses were performed using SPSS 20.0 (SPSS Inc., Chicago, IL, USA) software. A linear mixed model (LMM) in R was sued to evaluate the effect of sandy dune types on plant characteristics (including leaf, understorey herb biomass and litter C:N:P stoichiometry, DBH, SCS, H, SH, understorey grass coverage, and *S. moorcroftiana* coverage) and soil properties (BD, SM, DOC, AP, IN, and C:N:P stoichiometry) among different sampling sites, with “sandy dune type “ as the fixed factor and “sampling site” as the random factor, and the result was expressed as the mean ± standard error (SE). Pearson’s correlation analysis was performed to explore the stoichiometric relationships among plants, soils, and microbial biomass. Structural equation modeling (SEM) was used to determine the direct and indirect effects of soil physicochemical properties on the plant-soil-microbe C–N–P stoichiometry. Some variables (such as soil C:N and C:P ratios, leaf C:P ratio, understorey herb biomass C:P ratio, litter C:P and N:P ratios and microbial biomass C:N and C:P ratios) were excluded from SEM analysis due to insignificant effects or collinearity. The distributions of all data were evaluated and normality of data was determined using the Kolmogorov-Smirnov test, and the non-normal variables were log-transformed to improve normality, before conducting SEM analysis. We selected the best model based on overall goodness of fit parameters, including the χ^2^/df< 3, chi-square test statistic (*P* > 0.05), whole-model *P* value greater than 0.05, goodness of fit index (GFI> 0.90), comparative fit index (CFI > 0.90), and root mean square error of approximation (RMSEA< 0.05) (Schermelleh-Engel et al., 2003; Eisenhauer et al., 2016). SEM analyses were performed using AMOS version 24.0 software (IBM SPSS Inc.) and redundancy analysis (RDA) was conducted using CANOCO 5.0 tool to evaluate the relationships between C, N, and P concentrations and soil and leaf, understorey herb biomass, litter, and soil microbes. Origin 2021 software (Origin Lab Inc., Northampton, USA) was used to generate. *P*< 0.05 was considered statistically significant.

## 3 Results

### 3.1 Plant and soil properties

The results showed that plant and soil properties were significantly affected by sandy dune type, but exhibited differential responses (**Table 2**). Analysis of plant characteristics indicated that SBC and SH were high in the FD type, whereas DBH and SCS were higher in the MD type than in the other types (*P*< 0.05). Analysis of soil characteristics showed that the content of DOC, IN and AP increased from the MD to FD type; however, no significant changes were observed among sites in several cases. Soil pH, BD and SM did not significantly differ among the different sandy dune types. These findings indicate that sand dune stabilization and revegetation improve the soil fertility level.

**Table 2:** Characteristics of the plant and soil under three sandy dune types.

Types	FD	SFD	MD	F	*P*
Shrub coverage (%)	52.11 ± 2.62a	17.21 ± 1.43b	5.72 ± 0.36b	18.615	**<0.001**
Shrub crown size (m^2^)	1.19 ± 0.75c	1.78 ± 0.42b	1.96 ± 0.28a	33.236	**<0.001**
Diameter at breast height (cm)	9.88 ± 0.25c	9.93 ± 0.80b	12.55 ± 0.92a	13.843	**<0.001**
Shrub height (cm)	56.38 ± 2.50a	43.52 ± 5.32c	51.76 ± 3.13b	8.459	**0.001**
Soil moisture (%)	4.99 ± 0.54a	4.08 ± 0.29a	3.47 ± 0.29a	1.663	0.197
pH	9.93 ± 0.08a	9.60 ± 0.12a	9.58 ± 0.14a	1.918	0.154
BD (g cm^-3^)	1.52 ± 0.01a	1.55 ± 0.02a	1.56 ± 0.01a	1.074	0.347
DOC (mg kg^-1^)	93.34 ± 7.53a	37.79 ± 4.83a	30.84 ± 4.69a	1.275	0.286
Inorganic N (mg kg^-1^)	5.90 ± 0.39a	4.68 ± 0.76a	4.63 ± 0.49a	0.754	0.474
Available P (mg kg^-1^)	2.23 ± 0.16a	2.08 ± 0.15b	1.57 ± 0.11b	4.100	**0.021**

FD, fixed dune; SFD, semifixed dune; MD, moving dune; BD, soil bulk density; DOC, dissolved organic carbon. Different lowercase letters indicate significant difference among the sandy dune types at p< 0.05. P-values < 0.05 were marked in bold.

### 3.2 C:N:P stoichiometry in plants and soils

**Figure 1:**
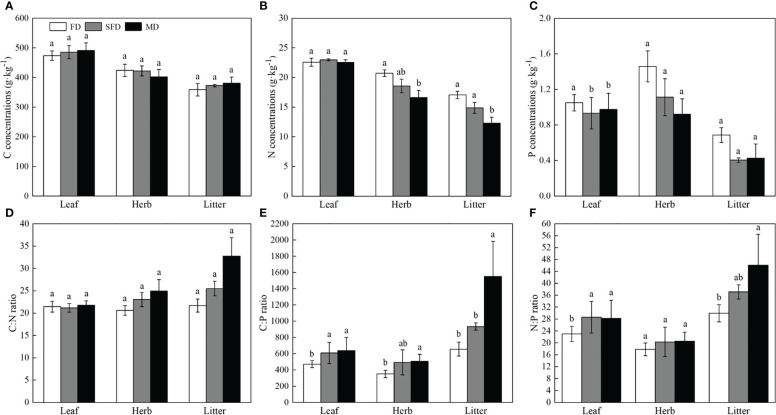
C, N, and P concentrations **(A–C)** and stoichiometry **(D–F)** in different components. *Sophora moorcroftiana* shrub under different sandy dune types. Different lowercase letters indicate significant difference under different sandy dune types based on analysis of variance and least significant difference tests (P< 0.05). FD, fixed dune; SFD, semifixed dune; MD, moving dune; Herb, understorey herb biomass.

Carbon concentrations in leaves, understorey herb biomass and litter were not significantly different among the three sandy dune types (*P* > 0.05; **Figure 1**). Nitrogen concentration was higher in leaves than in understorey herb biomass, and was significantly higher in understorey herb biomass and litter of the FD type than that in the MD (*P*< 0.05). The P concentration in leaves was significantly higher in the FD than that in leaves in the SFD and MD types, whereas the P concentrations in the understorey herb biomass and litter were highest in FD type compared with the other sandy dune types; however, no significant changes were observed among sites in several cases. The C:N in leaves, understorey herb biomass and litter were not significantly different among the three sandy dune types (*P* > 0.05) (**Figure 1**). The C:P ratio in leaves, understorey herb biomass and litter were lowest in FD type in the order MD > SFD > FD (*P*< 0.05) (**Figure 1**). The N:P ratio in leaves and litter in MD type was significantly higher than that in FD type (*P*< 0.05). The N:P ratio in understorey herb biomass was not significantly different among the three sandy dune types (**Figure 1**).

**Figure 2:**
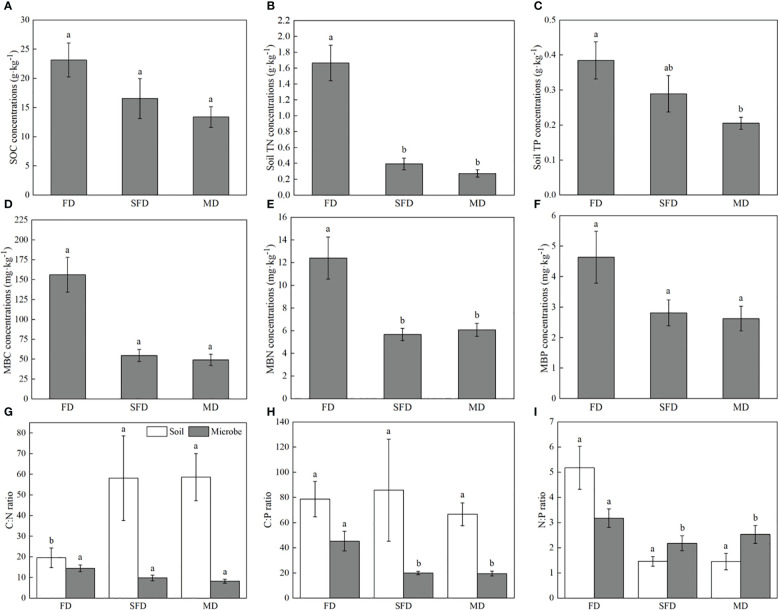
C, N, and P concentrations and stoichiometry in soils and microbes of Sophora moorcroftiana population in 0–20 cm soil layer under different sandy dune types. C, N, and P concentrations in soils **(A–C)** and microbes **(D–F)**. C, N, and P stoichiometry **(G–I)** in soils and microbes. Different lowercase letters indicate a significant difference under different sandy dune types (P < 0.05). FD, fixed dune; SGL, sandy gravel land; SFD, semifixed dune; MD, moving dune.

The sandy dune type significantly affected C, N and P concentrations and their ratios in soil and microbial biomass. however, sandy dune type did not affect soil C:P and N:P ratios. The C, N and P concentrations in the soil and microbial biomass increase with the increase in sand dune stabilization, and were significantly higher in the FD type than in MD type (**Figure 2B**). The soil C:N ratios in SFD and MD types were significantly relative to those in FD type (*P*< 0.05). The soil N:P ratio was higher in FD type than in SFD and MD types (*P* > 0.05). Microbial biomass C:P and N:P ratios were significantly higher in FD type than in SFD and MD types (*P*< 0.05).

### 3.3 Relationships between plant and soil C:N:P ecological stoichiometry and the potential regulators

The C, N, and P concentrations of plant components and microbial biomass and their stoichiometries exhibited different responses to soil C, N, and P concentrations and their ratios (**Figure S1**). Soil C concentrations were positively correlated with the C, N, and P concentrations in understorey herb biomass, litter, and microbial biomass and C concentration in litter (*P*< 0.05). Soil TN concentrations were positively correlated with N and P concentrations in understorey herb biomass, litter, and microbial biomass and C concentrations in understorey herb biomass and microbial biomass and negatively correlated with C concentration in leaves (*P*< 0.05). Soil P concentrations were positively correlated with C concentrations in leaves and microbial biomass and N and P concentrations in understorey herb biomass (*P*< 0.05). MBC, MBN and MBP were positively correlated with the C, N, and P concentrations in understorey herb biomass and litter. The findings showed no significant correlation between C, N, and P concentrations in leaves and C, N, and P concentrations in soil (**Figure S1**). Leaves C:N ratio was significantly correlated with soil C:N and N:P ratios. Understorey herb biomass C:P and N:P ratios were significantly correlated with soil C:P and N:P ratios. Litter C:N, C:P and N:P ratios were significantly correlated with soil C:N and N:P ratios. Microbial biomass C:N and C:P ratios were significantly correlated with soil C:N and N:P ratios (**Table 3**).

**Table 3:** Correlation coefficients between soil ecological stoichiometry and plant, soil microbial biomass ecological stoichiometry.

	Soil C:N	Soil C:P	Soil N:P
Leaf C:N	0.335**	0.190	-0.381**
Leaf C:P	0.178	-0.209	-0.268*
Leaf N:P	0.004	-0.170	-0.116
Herb C:N	0.273*	0.118	-0.082
Herb C:P	0.062	-0.258*	-0.315**
Herb N:P	-0.118	-0.393**	-0.294*
Litter C:N	0.456**	0.000	-0.413**
Litter C:P	0.464**	-0.009	-0.418*
Litter N:P	0.294*	-0.005	-0.354**
SMB C:N	-0.438**	0.137	0.534**
SMB C:P	-0.285*	0.149	0.318**
SMB N:P	-0.012	0.168	0.101

Herb, understorey herb biomass; SMB, soil microbial biomass. *P < 0.05, **P < 0.01.

**Figure 3:**
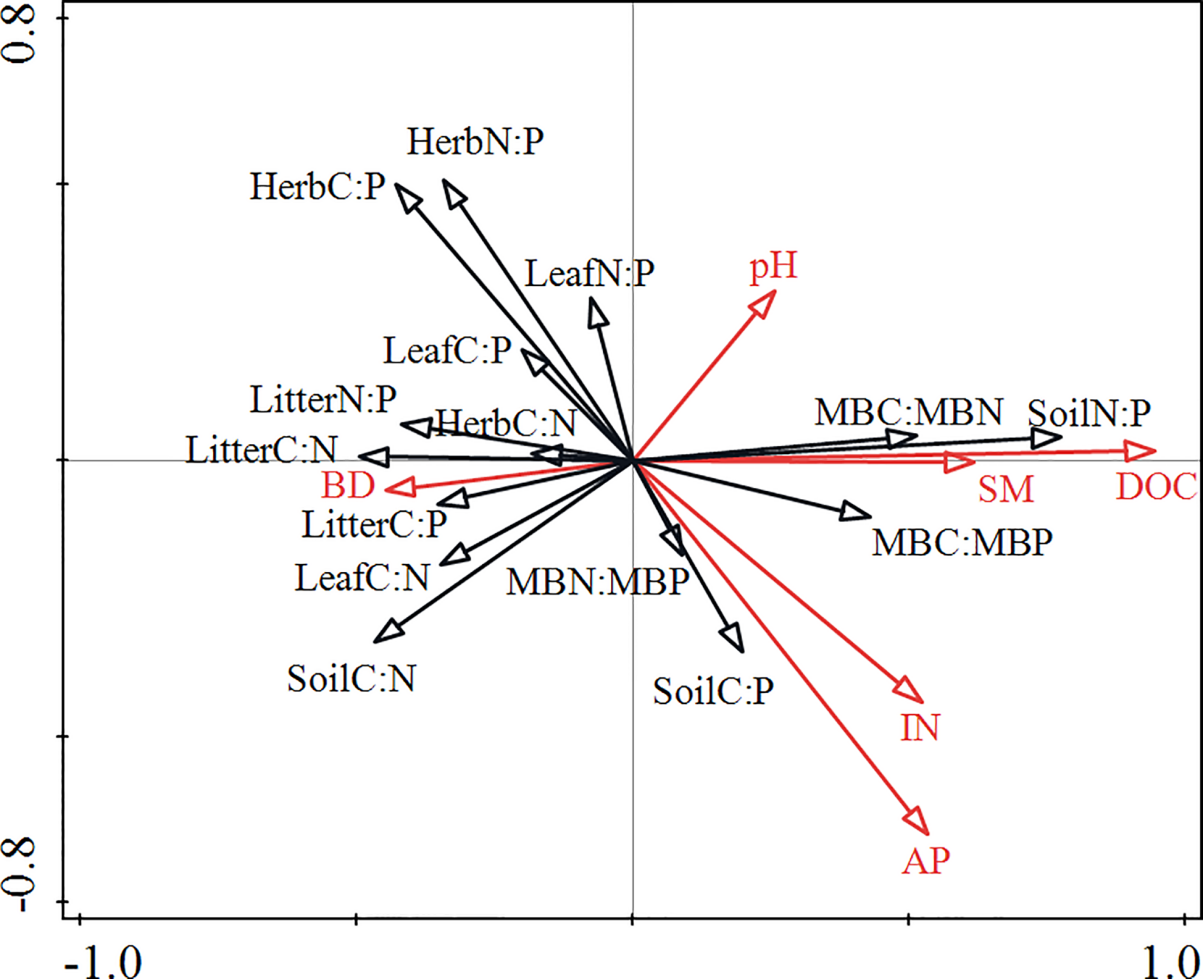
Redundancy analysis (RDA) showing the relationship between the plant-soil-microbe C:N:P ecological stoichiometry and soil physicochemical properties. SM, soil moisture content; IN, inorganic N (NH4+-N and NO3¯-N); AP, available phosphorus; BD, soil bulk density; DOC, dissolved organic carbon; Herb, understorey herb biomass.

RDA showed that the soil physicochemical properties (SM, BD, pH, DOC, AP, and IN) explained 30.7% of the total variation in the data, with axes 1 and 2 explaining 18.8% and 7.6% of the total variation, respectively (**Table 4**). DOC, AP and pH variables accounted for a greater proportion of the variance of the C:N:P ecological stoichiometry in plants, soils and microbes than SM, IN and BD variables (**Figure 3**). DOC and AP significantly affected C:N:P ecological stoichiometry in plants, soils and microbes.

**Table 4:** Redundancy analysis (RDA) of the plant-soil-microbe ecological stoichiometry and soil physicochemical properties using forward selection with a Monte Carlo permutation test.

Variables	Explains (%)	Contribution (%)	F-ratio	*P*-Value	Axis	1	2
DOC	16.8	54.6	14.2	0.001	Eigenvalues	18.8	7.6
AP	5.9	19.2	5.3	0.001	Explained variation	18.8	26.37
pH	2.9	9.5	2.7	0.031	Pseudo-canonical correlation	0.756	0.527
SM	2.8	9.0	2.6	0.028	Explained fitted variation	60.97	85.53
IN	1.6	5.2	1.5	0.187			
BD	0.7	2.4	0.7	0.627			

SM, soil moisture content; IN, inorganic N (NH4^+^-N and NO3^¯^-N); AP, available phosphorus; BD, soil bulk density; DOC, dissolved organic carbon.

**Figure 4:**
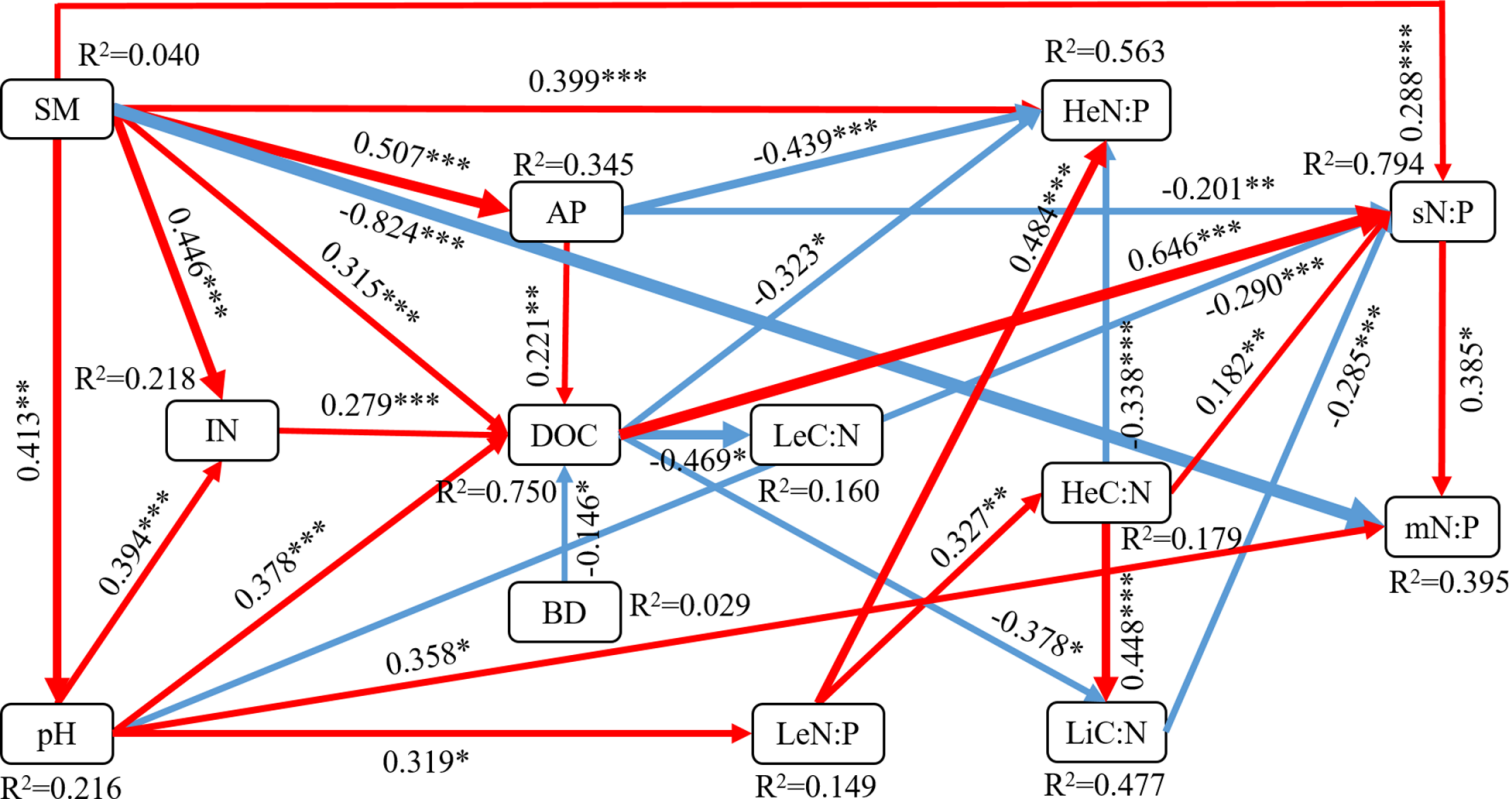
Structural equation modeling (SEM) analysis of the effects of soil physicochemical properties on the plant-soil-microbe C–N–P stoichiometry. Results of model fitting: χ2 = 0.004, df = 1, P = 0.953, GFI = 1.000, AGFI = 0.999, CFI = 1.000, RMSEA = 0.000. Numbers on arrows are standardized path coefficients. Red and blue arrows indicate positive and negative effects, respectively, with the thickness representing the extent of influence. R2 values represent the proportion of the variance explained for each endogenous variable. SM, soil moisture content; IN, inorganic N (NH4+-N and NO3¯-N); AP, available phosphorus; BD, soil bulk density; DOC, dissolved organic carbon; Le, Sophora moorcroftiana leaf; He, understorey herb biomass; Li, litter; s, soil; m, microbial biomass. Significance levels are as follows: *P < 0.05, **P < 0.01, and ***P < 0.001.

SEM exhibited a good fit according to the χ^2^ test, GFI, AGFI, CFI and RMSEA metrics (**Figure 4**). SEM analysis showed that soil physicochemical properties directly affected microbe stoichiometry or indirectly through plant and soil stoichiometry. SM had the greatest negative direct effect on microbial biomass N:P ratio (path coefficient = −0.824) compared with the other five variables and was significantly positively correlated with understorey herb biomass N:P ratio (0.399). SM indirectly impacted on leaves C:N and N:P ratios. pH indirectly impacted on N:P ratios in leaves, soil and microbial biomass. The results showed that AP directly affected N:P ratios in understorey herb biomass and soil. DOC directly affected N:P ratios in understorey herb biomass and soil and C:N ratios in leaves and litter. The findings showed that IN indirectly affected plant and soil stoichiometry through DOC. Notably, BD was mainly modulated plant and soil stoichiometry through DOC. Soil N:P ratio directly affected microbial biomass C:N and N:P ratios. Leaf N:P ratio directly affected microbial biomass N:P ratio through C:N ratios in understorey herb biomass and litter as well as soil N:P ratio.

### 3.4 Effect of shrub age on plant and soil C:N:P ecological stoichiometry

**Figure 5:**
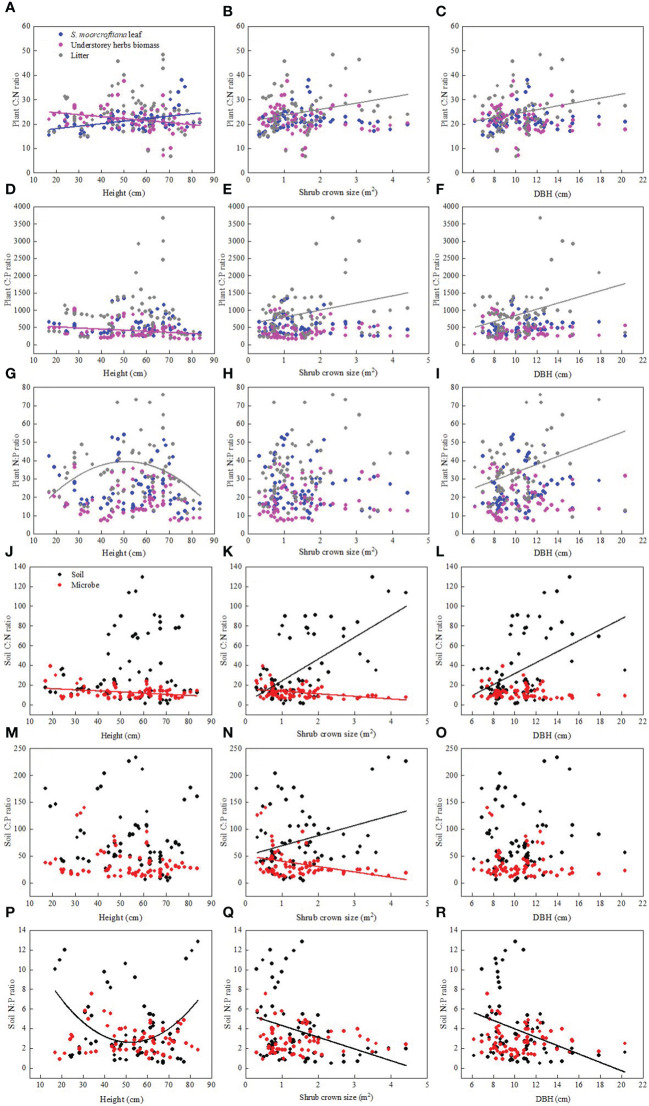
Relationships between Sophora moorcroftiana height and plant stoichiometry **(A–G)**, shrub crown size and plant stoichiometry **(B–H)** diameter at breast height (DBH) and plant stoichiometry **(C–I)**, Sophora moorcroftiana height and soil stoichiometry **(J–P)**, shrub crown size and soil stoichiometry **(K–Q)**, diameter at breast height (DBH) and soil stoichiometry **(L–R)**, respectively.

Shrub age characteristics (including diameter at breast height, height and shrub crown size) exhibited varying responses to soil and leaf C:N:P stoichiometry (**Figure 5**). The height of *S. moorcroftiana* was significantly negatively correlated with C:N and C:P ratios of understorey herb biomass (**Figure 5**). On the contrary, height of *S. moorcroftiana* was significantly positively correlated with the C:N ratio of leaves (**Figure 5**) and exhibited a downward “unimodal” trend with litter N:P ratio (**Figure 5**). Soil N:P ratio decreased from 17 to 50 cm height and then increased, exhibiting an upward “unimodal” trend (**Figure 5**). Diameter at breast height and shrub crown size were positively correlated with C:N and C:P ratios of litter and N:P ratio of litter (**Figures 5B, C, E**). Conversely, diameter at breast height and shrub crown size were significantly negatively correlated with soil N:P (**Figure 5**), but significantly positively correlated with soil C:N ratio (**Figures 5**). Shrub crown size was significantly negatively correlated with microbial biomass C:N and C:P ratios (**Figures 5**). These findings indicate that the C:N:P ratio in soil was significantly correlated with the shrub crown size and diameter at breast height.

## 4 Discussion

### 4.1 C:N:P stoichiometry in plants among the different sandy dune types

N and P are the main mineral elements that limit plant growth. The two mineral elements play important roles in modulating plant growth, reproduction and metabolism in most terrestrial ecosystems (Chapin, 1980; Reich et al., 1997; Han et al., 2005). Previous studies reported that leaf N:P ratio in plant biomass indicates the relative N or P limitation at the community level. Shifts in limitation cause changes in plant functional traits, vegetation composition and botanical diversity (Koerselman and Meuleman, 1996; Roem and Berendse, 2000; Güsewell, 2004). The “law of the minimum” states that there is a “critical N:P ratio” below which plant growth is limited only by N, and conversely above which growing plants are only limited by P (Güsewell, 2004). Previous findings indicate that N:P ratios > 16 indicate P limitation, whereas N:P ratios< 14 represent N limitation, and plant growth is co-limited by N and P under intermediate values (Aerts and Chapin, 2000; Reich, 2005; Fan et al., 2015). Güsewell (2004) observed that plant growth was relatively limited by nitrogen when the N:P ratio in leaves was< 10, whereas plant growth was relatively limited by phosphorus when the N:P ratio was > 20. Moreover, plant growth was limited by N or P when the N:P ratio was between 10 and 20, depending on plant-specific conditions.

In the current study, the leaf N:P ratio ranged from 18.5 to 28.3, and the mean foliar N:P ratio was 25.7, higher than the value reported by Han et al. (2005). The average N:P ratio of China’s flora is 16.3 and the global averages are 12.7 and 13.8 (Elser et al., 2000; Reich and Oleksyn, 2004). Leaf N concentrations ranged between 22.5 to 23.0 g·kg^−1^, and the P concentrations were > 1.0 mg g^-1^, ranging from 1.0 to 1.3 g·kg^−1^ (**Figure 1**). This finding indicates that P was the main limiting element for the *S. moorcroftiana* population in the study area. This finding is consistent with a report that Chinese vegetation exhibits a higher degree of P limitation, as indicated by analysis of 753 species (Han et al., 2005). The high leaf N:P ratios (low P content) can be attributed to the low P content of soils in China (Han et al., 2005). Plant and soil phosphorus are generally coupled at the ecosystem scale (Aerts and Chapin, 2000; Han et al., 2005). In addition, the P limitation may be caused by the ability of leguminous plants to fix N from the air resulting in a higher N:P ratio (Yang et al., 2018), whereas P is a ‘rock-derived’ element and cannot be obtained in high amounts from the soil (Walker and Syers, 1976). Recent studies report that understorey species have higher ability to obtain P from the soil than dominant tree species (Huang et al., 2013), resulting in P limitation in overstorey trees (Zhang et al., 2019). Roots of overstory trees are distributed deeper into the soil as they age (Jiao et al., 2016). Deep-rooted systems cannot preferentially access P and other resources supplied by plants (Chen et al., 2016a), resulting in lower P concentrations and higher N:P ratio in leaves (**Figure 1**), and which limits the growth of *S. moorcroftiana* (**Table 2**).

Litter is a nutrient pool (Xiao et al., 2015; Liu and Wang, 2021), and its C:N:P stoichiometry ratio modulates soil nutrient availability and quality in terrestrial ecosystems (Villalobos-Vega et al., 2011; Zhang et al., 2017). Previous studies reported that a litter C:N ratio< 40, promotes decomposition of the litter and net release of nitrogen, and lower ratios of C:N and C:P are associated with faster decomposition rate (Parton et al., 2007). In this study, the C:N ratio in litter was lower than 40, indicating a high rate of decomposition and faster release of nitrogen from litter. N:P ratio is a vital indicator for characterizing the decomposition rate of litter, and a lower ratio is associated with higher rate of litter decomposition (Sun et al., 2019). The present results showed that the N:P ratio in litter ranged from 29.9 to 46.1 and higher than the level of 25 reported previously (Güsewell and Verhoeven, 2006). This N:P ratio in litter indirectly indicated the nutrient limitations of *S. moorcroftiana* shrub community growth in the study area.

### 4.2 C:N:P stoichiometry in soils and microbes across the different sandy dune types

Vegetation restoration can affect plant diversity and composition (Zhao et al., 2019), increasing quantity and quality of litter and rhizodeposition (Zhang et al., 2019). These effects ultimately improve the soil pool and soil nutrients (Deng et al., 2013; Yang et al., 2018; Deng et al., 2018). Variations in environmental conditions of soil structure, pH, temperature and moisture can be modulated by vegetation restoration (Fernández-Calviño et al., 2011; Ahmed et al., 2019), which in turn increases soil microbial biomass and enzyme activities associated with nutrient decomposition and circulation (Zhang et al., 2019; **Figure 2**). Ultimately, these effects increase soil C, N, and P contents (**Figure 2**). An increase in C, N and P imbalance causes changes in soil C:N:P stoichiometry (Zhang et al., 2019). In the present study, the soil C:N ratios of the SFD and MD types were significantly higher than the rations in FD type. The N:P ratios varied in an opposite pattern among the three sandy dune types (**Figure 2**), because the increasing ratios of N was higher than that of C and P (**Figure 2**). Soil C:N ratio is an important indicator of microbial activity. Soil C:N ratio determines the mineralization rate of SOM and release of available N, and can directly modulate the competition between microorganisms and plants for N and indirectly affect plant photosynthesis (Brust, 2019; Qi et al., 2022). The average soil C:P ratio in this study was 77.3, which is higher than the average ratio reported in China (61.0), and the N:P ratio (3.8) was lower than the global average ratio (5.9) (Han et al., 2005; Yang et al., 2018). This finding indicates that the soil N (C:N = 36.8) and P (C:P = 62.5) (Han et al., 2005) contents in the study sites were relatively low. This observation can be attributed to insufficient supply of P caused by the N fixation process (Bell et al., 2014).

**Figure 6:**
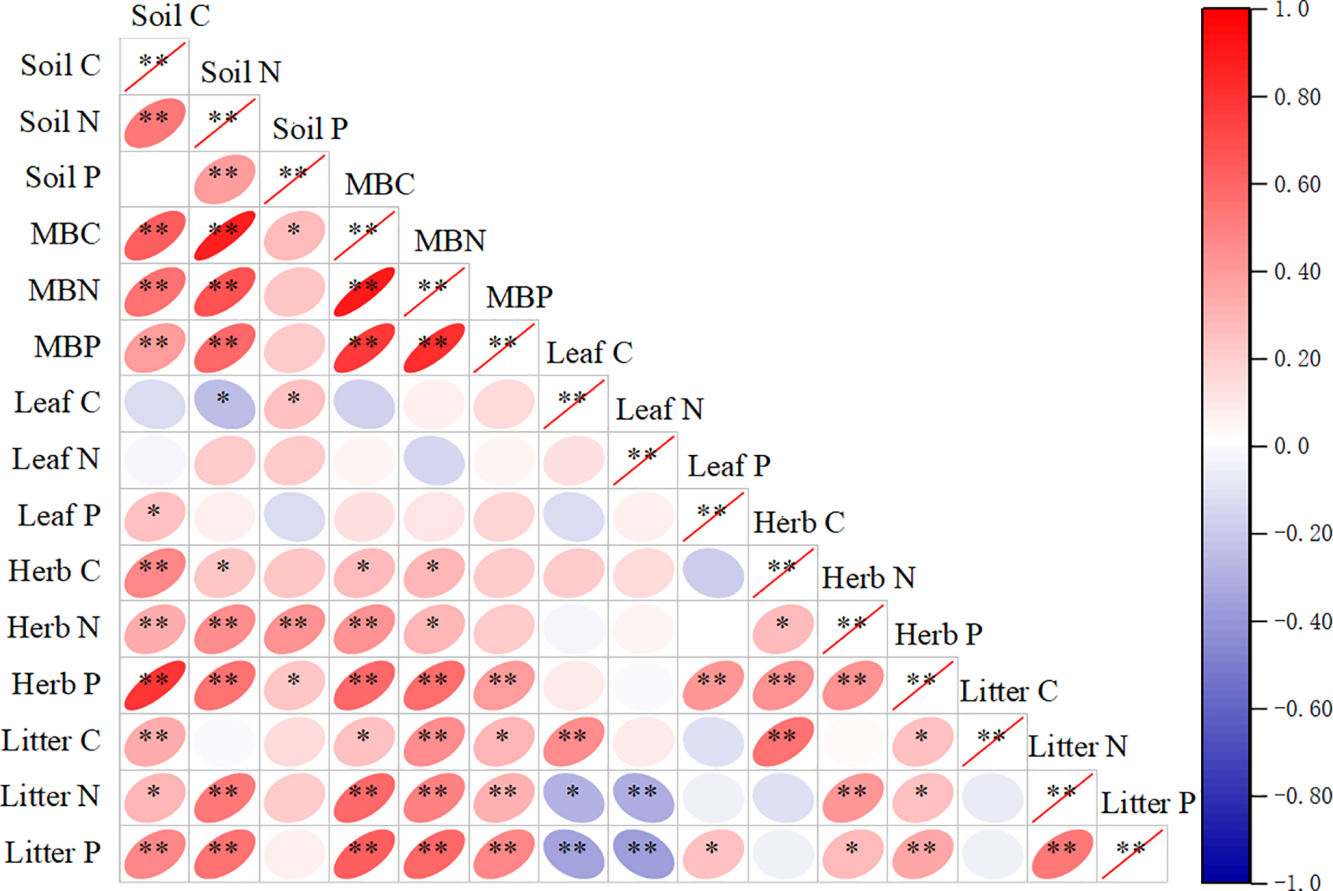
Correlations between soil, soil microbial biomass, Sophora moorcroftiana leaf, herb, and litter C, N, and P concentrations. Herb, understorey herb biomass. *P < 0.05, **P < 0.01.

Soil microbes act as the main nutrient reservoir, and their stoichiometry is highly correlated with the above-ground plant communities (Qi et al., 2022). Soil microbes can maintain stoichiometric homeostasis through compensatory regulation (Chen and Chen, 2021). The soil microbial biomass C:N:P stoichiometry in this study was 15:1.2:1, which was narrower than that of shrub-land (43:6:1), desert (31:4:1) and the global average (42:6:1) (Xu et al., 2013). This large discrepancy is mainly attributed to the lower SOC content in deserts (Wang et al., 2022b); The stoichiometric variability decreases with soil OM processing (Mooshammer et al., 2014). In this study, significantly positive correlations were observed between the microbial biomass C, N, and P and SOC (**Figure S1**). Soil microbial biomass C is mainly positively correlated with microbial biomass N (**Figure 6**), as higher microbial biomass leads to higher microbial N requirements (Deng et al., 2016). A meta-analysis by Xu et al. (2013) reported that the average microbial biomass C:N ratios was 7.6 and ranged from 4.5 to 12.5, and the average of microbial N:P ratios was 5.6, with a rang from 3.5 to 10.6. The soil samples at the present study site exhibited significantly higher microbial biomass C:N (13.4) and lower N:P (2.9) ratio (**Figure 2**), which can be attributed to relatively lower N availability to soil microbes. A greater microbial biomass C:N ratio indicates a lower level of N in the soil and poor mineralization rate due to a lack of enough detrital material and the desired type of microbial population (Arunachalam and Pandey, 2002).

### 4.3 Correlations with the C:N:P stoichiometry

In this study, plant C and N concentrations were significantly correlated with soil C and N concentrations but weakly correlated with soil P concentrations (**Figure S1**). This finding indicates that the C and N contents in understorey herb plants and litter have a significant relationship with soil nutrients. This type of interaction is observed mainly because the soil C content is determined by the coefficient of humus and organic matter in the soil, whereas the N in decomposed litter is mainly absorbed to the soil, whereas P is mainly retained in the litter (Robbins et al., 2019). The soil C:N ratio was significantly correlated with the C:N ratios in leaves, litter and microbial biomass (**Table 3**). The litter in the surface layer can act as the main supply to the soil, leading to accumulation of C and N (Yang and Liu, 2019). Nitrogen-fixing plants effectively absorb N and SOC (Li et al., 2014). The effects of diversity on plant C:N:P ratios may modulate soil nutrient input levels and microorganisms through litter (Zechmeister-Boltenstern et al., 2015; Chen and Chen, 2021). RDA indicated that soil physicochemical properties explained the largest variance in the plant and soil C:N:P stoichiometry (**Figure 3**), with DOC, AP, pH and SM as the most important factors (**Table 4**). Water loss can lead to nutrient loss (Peng et al., 2020), which is consistent with the observed significant positive correlations between the SM and DOC, AP and IN in this study. Plant diversity positively affects soil microbial community through the regulation of water absorption (Wang et al., 2022a). The present results showed a positive correlation between SM and N:P ratios in the understorey herb biomass and soil, and a negative correlation between SM and MBN : MBP (**Figure 4**). Soil pH is a key indicator of soil nutrient availability and is correlated with soil microbial activity. Soil pH and litter elements ratios are the main factors that modulate surface soil N:P ratio (Yang et al., 2018; Qiao et al., 2020), which is consistent with the present findings. Soil C:N:P ratios represent stoichiometric ratios of their sources, including plant and microbial residues (Chen and Chen, 2021). Higher litter inputs increase soil nutrient retention, resulting in reduction of soil microbial C: nutrient ratio (Chen and Chen, 2021). A previous study reported that slow-growing shrubs in P-poor systems transfer P from deeper soil layers to shallow soils, which helps meet the high leaf P requirements of fast-growing understorey herbs (He et al., 2014; Luo et al., 2021). This explains why leaf P content was closely correlated with the understorey herb biomass P content and the C:P and N:P ratios in the present study. This finding indicates that a correlation between the P levels of plants and soil P levels. AP can indicate the degree of P limitation (Zhang et al., 2019). These results validated our second hypothesis that soil properties is a key driver of C:N:P stoichiometry in plant-soil-microbe systems.

## 5 Conclusion

The aim of this study was to explore the effects of sandy dune type on the C:N:P stoichiometry in “plant-soil-microbe” and evaluate the factors that modulate *S. moorcroftiana* shrub community. The findings showed that sandy dune type had significant effects on C, N, and P contents and stoichiometry in plants, soils and microbes, implying that the C:N:P stoichiometric ratios can form a complex network in this region. The results indicated that a significant correlation was observed between plants and soils C:N:P stoichiometry. This observation implies that the changes in C:N:P stoichiometry may be induced by shifts in soil physicochemical properties, especially DOC, AP, SM and pH. Furthermore, significant increases in plant and soil C:P ratios and decreases in soil N:P ratios were observed with an increase in shrub age, indicating that the demand for P increased with shrub age. In this study, C:N:P stoichiometry interactions among plants, soils, and soil microbes and their responses to the sandy dune type were evaluated. The findings highlight the importance of soil physicochemical properties in shaping the C:N:P stoichiometry and improve our understanding of the nutrient stoichiometry patterns and management strategies of plants and soils in alpine desert ecosystems on the Tibetan Plateau.

## Data availability statement

The original contributions presented in the study are included in the article/**Supplementary Material**. Further inquiries can be directed to the corresponding authors.

## Author contributions

RD: Formal analysis, Writing-original draft, Writing-review and editing, SY: Resources, Funding acquisition, XW: Funding acquisition, LX: Resources, YM: Conceptualization, Resources, YW: Resources, LZ: Investigation, MZ: Investigation, JQ: Investigation. All authors contributed to the article and approved the submitted version.
