# Supplementary material for: C:N:P stoichiometry in plant, soil and microbe in Sophora moorcroftiana shrubs across three sandy dune types in the middle reaches of the Yarlung Zangbo River
Source: Front Plant Sci. 2023 Jan 11;13:1060686. doi: 10.3389/fpls.2022.1060686 (PMC9874299; doi:10.3389/fpls.2022.1060686)
Supplement: Supplementary file 1 [file DataSheet_1.docx]

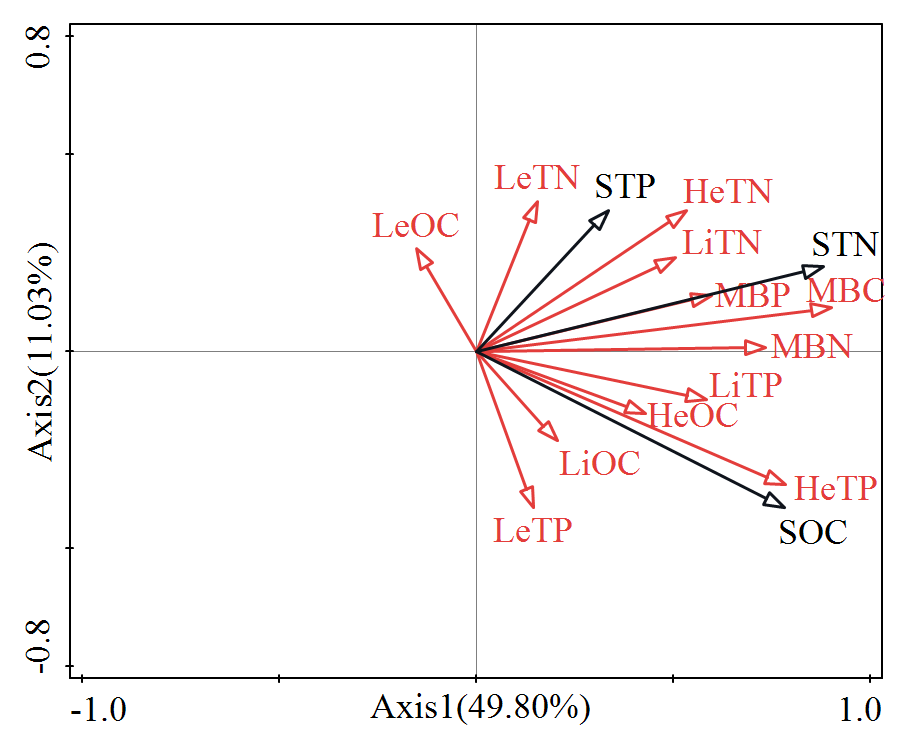


**Figure S1.** Redundancy analysis (RDA) showing the relationship between soil, *Sophora moorcroftiana* leaf, litter, understorey herb biomass, and soil microbial biomass C, N, P concentrations. SOC: soil organic carbon concentration, STN: soil total nitrogen concentration, STP: soil total phosphorus concentration, LeOC: *Sophora moorcroftiana* leaf organic carbon concentration, LeTN: *Sophora moorcroftiana* leaf total nitrogen concentration, LeTP: *Sophora moorcroftiana* leaf total phosphorus concentration, LiOC: litter organic carbon concentration, LiTN: litter total nitrogen concentration, LiTP: litter total phosphorus concentration, HeOC: understorey herb biomass organic carbon concentration, HeTN: understorey herb biomass total nitrogen concentration, HeTP: understorey herb biomass total phosphorus concentration, MBC: soil microbial biomass carbon concentration, MBN: soil microbial biomass nitrogen concentration, MBP: soil microbial biomass phosphorus concentration.

**Table S1** Types and indicators of aeolian sandy land in the middle reaches of the Yarlung Zangbo River basin.

| Indicators | Wind-deposited sand land | | |
| --- | --- | --- | --- |
|  | Fixed dunes | Semi-fixed dunes | Moving dunes |
| Surface feature | Flat sand land, no obvious wind erosion, surface environment is stable or basically stable | Flat sand land, the distribution is relatively uniform, but there is still a more obvious sand movement. | Sand movement, mountain slope sand land, barchan dune and dune chain, complex dune |
| Vegetation cover | ≥ 30% | 10–30% | < 10% |

| Parameter | SOC | STN | STP | SCN | SCP | SNP | SMBC | SMBN | SMBP | SMBCN | SMBCP | SMBNP | LeOC | LeTN | LeTP | LeCN | LeCP | LeNP | LiOC | LiTN | LiTP | LiCN | LiCP |
| --- | --- | --- | --- | --- | --- | --- | --- | --- | --- | --- | --- | --- | --- | --- | --- | --- | --- | --- | --- | --- | --- | --- | --- |
| SOC | 1 | 0.525** | 0.016 | 0.014 | 0.668** | 0.569** | 0.629** | 0.559** | 0.389* | 0.265* | 0.387** | 0.341** | -0.122 | -0.022 | 0.256* | -0.055 | -0.206 | -0.191 | 0.330* | 0.280* | 0.468** | -0.044 | -0.197 |
| STN |  | 1 | 0.390** | -0.647** | 0.095 | 0.809** | 0.864** | 0.663** | 0.585** | 0.448** | 0.347* | 0.117 | -0.248* | 0.203 | 0.064 | -0.333** | -0.228 | -0.082 | -0.006 | 0.527** | 0.546** | -0.409* | -0.475** |
| STP |  |  | 1 | -0.384** | -0.533** | -0.149 | 0.278* | 0.230 | 0.211 | 0.084 | 0.105 | 0.020 | 0.254* | 0.206 | -0.123 | 0.051 | 0.057 | 0.086 | 0.158 | 0.213 | 0.078 | -0.066 | -0.142 |
| SCN |  |  |  | 1 | 0.362** | -0.501** | -0.459** | -0.252* | -0.295* | -0.438** | -0.285* | 0.012 | 0.124 | -0.286* | 0.043 | 0.335** | 0.178 | 0.004 | 0.240* | -0.342** | -0229 | 0.456** | 0.464** |
| SCP |  |  |  |  | 1 | 0.471* | 0.211 | 0.183 | 0.084 | 0.137 | 0.149 | 0.168 | -0.340** | -0.076 | 0.302** | -0.190 | -0.209 | -0.170 | 0.174 | 0.070 | 0.139 | 0.000 | -0.009 |
| SNP |  |  |  |  |  | 1 | 0.708** | 0.501** | 0.441** | 0.534** | 0.318** | 0.101 | -0.433** | 0.071 | 0.107 | -0.381** | -0.268* | -0.116 | -0.129 | 0.421** | 0.471** | -0.413** | -0.418** |
| SMBC |  |  |  |  |  |  | 1 | 0.884** | 0.764** | 0.262* | 0.258** | 0.153 | -0.160 | 0.055 | 0.123 | -0.140 | -0.215 | -0.161 | 0.257* | 0.582** | 0.622** | -0.286* | -0.417** |
| SMBN |  |  |  |  |  |  |  | 1 | 0.810** | -0.159 | -0.012 | 0.178 | 0.062 | -0.148 | 0.105 | 0.179 | -0.099 | -0.177 | 0.449** | 0.482** | 0.584** | -0.081 | -0.299* |
| SMBP |  |  |  |  |  |  |  |  | 1 | -0.081 | -0.294** | -0.343** | 0.149 | 0.055 | 0.171 | 0.053 | -0.143 | -0.176 | 0286 | 0.315** | 0.478** | -0.084 | -0.309** |
| SMBCN |  |  |  |  |  |  |  |  |  | 1 | 0.584** | 0.012 | -0.501** | 0.180 | -0.071 | -0.484** | -0.170 | 0.051 | -0.413** | 0.310** | 0.203 | -0.497** | -0.358** |
| SMBCP |  |  |  |  |  |  |  |  |  |  | 1 | 0.744** | -0.491** | 0.078 | 0.057 | -0.369** | -0.196 | -0.063 | -0.229 | 0.264* | 0.259* | -0.325** | -0.218 |
| SMBNP |  |  |  |  |  |  |  |  |  |  |  | 1 | -0.265* | -0.224 | 0.022 | 0.039 | -0.060 | -0.090 | 0.079 | 0.148 | 0.206 | -0.015 | 0.020 |
| LeOC |  |  |  |  |  |  |  |  |  |  |  |  | 1 | 0.132 | -0.140 | 0.571** | 0.351** | 0.154 | 0.446** | -0.283* | -0.344** | 0.482** | 0.340** |
| LeTN |  |  |  |  |  |  |  |  |  |  |  |  |  | 1 | 0.080 | -0.718** | -0.088 | 0.209 | 0.083 | -0.311* | -0.378** | 0.228 | 0.259* |
| LeTP |  |  |  |  |  |  |  |  |  |  |  |  |  |  | 1 | -0.222 | -0.883** | -0.881** | -0.104 | -0.050 | 0.259* | -0.106 | -0.238* |
| LeCN |  |  |  |  |  |  |  |  |  |  |  |  |  |  |  | 1 | 0.366** | -0.012 | 0.245* | 0.103 | 0.087 | 0.129 | 0.024 |
| LeCP |  |  |  |  |  |  |  |  |  |  |  |  |  |  |  |  | 1 | 0.918** | 0.303** | -0.099 | -0.260* | 0.323** | 0.270* |
| LeNP |  |  |  |  |  |  |  |  |  |  |  |  |  |  |  |  |  | 1 | 0.227 | -0.129 | -0.326** | 0.276* | 0.273* |
| LiOC |  |  |  |  |  |  |  |  |  |  |  |  |  |  |  |  |  |  | 1 | -0.065 | -0.044 | 0.620** | 0.332** |
| LiTN |  |  |  |  |  |  |  |  |  |  |  |  |  |  |  |  |  |  |  | 1 | 0.530** | -0.785** | -0.567** |
| LiTP |  |  |  |  |  |  |  |  |  |  |  |  |  |  |  |  |  |  |  |  | 1 | -0.403** | -0.700** |
| LiCN |  |  |  |  |  |  |  |  |  |  |  |  |  |  |  |  |  |  |  |  |  | 1 | 0.691** |
| LiCP |  |  |  |  |  |  |  |  |  |  |  |  |  |  |  |  |  |  |  |  |  |  | 1 |

**Table S2** Pearson correlation matrix among soil physical-chemical properties, plant and soil C:N:P stoichiometric characteristics, and plant community characteristics

Table S2 continued

| Parameter | LiNP | HeOC | HeTN | HeTP | HeCN | HeCP | HeNP | SM | pH | BD | DOC | NH4+ | NO3- | AP | SC | SCS | DBH | SH |
| --- | --- | --- | --- | --- | --- | --- | --- | --- | --- | --- | --- | --- | --- | --- | --- | --- | --- | --- |
| SOC | -0.271* | 0.462** | 0.329* | 0.790** | 0.113 | -0.491* | -0.672** | 0.425** | 0.092 | -0.189 | 0.572** | 0.594** | 0.317** | 0.690** | 0.271* | -0.095 | -0.157 | 0.098 |
| STN | -0.433** | 0.234* | 0.452** | 0.542** | -0.174 | -0.390** | -0.338** | 0.510** | 0.159 | -0.421** | 0.780** | 0.797** | 0.209 | 0.425** | 0.435** | -0.427** | -0.364** | 0.079 |
| STP | -0.134 | 0.224 | 0.422** | 0.234* | -0.128 | -0.151 | -0.093 | -0.174 | -0.005 | -0.040 | 0.106 | 0.097 | -0.040 | 0.024 | 0.390** | -0.348** | -0.174 | 0.117 |
| SCN | 0.294* | -0.006 | -0.316** | -0.123 | 0.273* | 0.062 | -0.118 | -0.225 | -0.192 | 0.241* | -0.433** | -0.433** | 0.124 | -0.033 | -0.446** | 0.627** | 0.450** | 0.191 |
| SCP | -0.005 | 0.115 | -0.045 | 0.361** | 0.118 | -0.258* | -0.393** | 0.360** | -0.115 | -0.086 | 0.271* | 0.260* | 0.297* | 0.426** | -0.120 | 0.304** | 0.092 | -0.043 |
| SNP | -0.354** | 0.119 | 0.199 | 0.415** | -0.082 | -0.315** | -0.294* | 0.679** | 0.140 | -0.406** | 0.747** | 0.725** | 0.247* | 0.444** | 0.224 | -0.331* | -0.337** | -0.089 |
| SMBC | -0.421** | 0.276* | 0.432** | 0.581** | -0.104 | -0.381** | -0.393** | 0.562** | 0.195 | -0.365** | 0.815** | 0.880** | 0.362** | 0.475** | 0.371** | -0.222 | -0.239* | 0.296* |
| SMBN | -0.402** | 0.188** | 0.283* | 0.579** | 0.024 | -0.303** | -0.377** | 0.438** | 0.109 | -0.222 | 0.608** | 0.701** | 0.332** | 0.373** | 0.378** | -0.069 | -0.182 | 0.451** |
| SMBP | -0.379** | 0.204 | 0.210 | 0.398** | -0.006 | -0.170 | -0.213 | 0.581** | 0.162 | -0.112 | 0.637** | 0.655** | 0.270* | 0.356* | 0.215 | -0.064 | -0.153 | 0.402** |
| SMBCN | -0.170 | 0.041 | 0.319** | 0.070 | -0.231 | -0.195 | -0.087 | 0.313** | 0.277* | -0.202 | 0.476** | 0.312** | -0.001 | 0.298* | 0.109 | -0.385** | 0.182 | -0.312** |
| SMBCP | -0.117 | 0.108 | 0.346** | 0.280* | -0.163 | -0.313** | -0.283* | -0.095 | 0.249* | -0.239* | 0.357** | 0.262* | 0.027 | 0.208 | 0.313 | -0.332** | -0.168 | -0.201 |
| SMBNP | -0.042 | 0.142 | 0.172 | 0.313** | 0.010 | -0.275* | -0.328** | -0.262* | 0.065 | -0.195 | 0.070 | 0.084 | 0.041 | 0.060 | 0.285* | -0.115 | -0.081 | -0.017 |
| LeOC | 0.206 | 0.201 | -0.039 | 0.092 | 0.219 | 0.133 | -0.030 | -0.287* | -0.284* | 0.231 | -0.369** | -0.239 | 0.039 | -0.121 | 0.163 | 0.061 | 0.097 | 0.358** |
| LeTN | 0.261* | 0.156 | 0.056 | -0.009 | 0.096 | 0.113 | 0.067 | 0.014 | -0.097 | -0.110 | 0.054 | 0.200 | -0.068 | -0.053 | -0.038 | -0.088 | -0.066 | -0.178 |
| LeTP | -0.246* | -0.182 | 0.000 | 0.418** | -0.218 | -0.476** | -0.393** | 0.129 | -0.230 | 0.107 | 0.055 | 0.218 | 0.184 | 0.227 | -0.095 | 0.258* | 0.075 | -0.054 |
| LeCN | -0.066 | 0.028 | -0.008 | 0.061 | 0.043 | 0.000 | -0.025 | -0.214 | -0.063 | 0.247* | -0.274* | -0.333** | 0.061 | -0.025 | 0.179 | 0.101 | 0.117 | 0.422** |
| LeCP | 0.184 | 0.171 | -0.142 | -0.419** | 0.333** | 0.575** | 0.426** | -0.194 | 0.187 | 0.006 | -0.153 | -0.330** | -0.059 | -0.210 | 0.087 | -0.096 | -0.050 | 0.127 |
| LeNP | 0.230 | 0.197 | -0.087 | -0.456* | 0.305** | 0.596** | 0.468** | -0.101 | 0.240* | -0.112 | -0.043 | -0.214 | -0.125 | -0.208 | 0.057 | -0.167 | -0.104 | -0.030 |
| LiOC | 0.113 | 0.555** | 0.028 | 0.256** | 0.489** | 0.195 | -0.121 | -0.034 | -0.111 | -0.004 | -0.016 | 0.168 | 0.170 | 0.072 | -0.102 | 0.208 | 0.143 | 0.300* |
| LiTN | -0.268* | -0.108 | 0.418** | 0.258* | -0.420** | -0.274* | -0.072 | 0.362** | 0.278* | -0.183 | 0.561** | 0.473** | 0.152 | 0.383** | 0.423** | -0.185 | -0.203 | 0.246* |
| LiTP | -0.777** | -0.054 | 0.271* | 0.343** | -0.284* | -0.283* | -0.166 | 0.480** | 0.267* | -0.152 | 0.640** | 0.558** | 0.309** | 0.458** | 0.245* | -0.068 | -0.144 | 0.190 |
| LiCN | 0.287* | 0.364** | -0.334** | -0.086 | 0.609** | 0.330** | -0.001 | -0.281* | -0.232* | 0.139 | -0.423** | -0.278* | -0.018 | -0.243* | -0.373** | 0.277* | 0.250* | 0.062 |
| LiCP | 0.853** | 0.185 | -0.274* | -0.179 | 0.403** | 0.218 | 0.009 | -0.316** | -0.216 | 0.137 | -0.452** | -0.332** | -0.163 | -0.259* | -0.389** | 0.285* | 0.352** | 0.078 |
| LiNP | 1 | 0.012 | -0.133 | -0.207 | 0.139 | 0.129 | 0.064 | -0.313** | -0.175 | 0.124 | -0.391** | -0.325** | -0.213 | -0.229 | -0.283* | 0.190 | 0.329** | -0.028 |
| HeOC |  | 1 | 0.266* | 0.436** | 0.630** | 0.036 | -0.421** | -0.071 | 0.063 | -0.203 | 0.118 | 0.250* | -0.172 | 0.076 | -0.036 | -0.297* | -0.197 | -0.129 |
| HeTN |  |  | 1 | 0.432** | -0.562** | -0.494** | -0.278* | 0.106 | 0.251* | -0.202 | 0.462** | 0.377** | -0.097 | 0.304** | 0.405** | -0.174 | 0.058 | 0.237* |
| HeTP |  |  |  | 1 | -0.009 | -0.758** | -0.871** | 0.192 | -0.103 | -0.171 | 0.399** | 0.536** | 0.136 | 0.587** | 0.379** | -0.177 | -0.115 | 0.177 |
| HeCN |  |  |  |  | 1 | 0.458** | -0.109 | -0.106 | -0.133 | -0.011 | -0.260* | -0.095 | -0.040 | -0.179 | -0.373** | -0.129 | -0.200 | -0.276* |
| HeCP |  |  |  |  |  | 1 | 0.817** | -0.165 | 0.075 | 0.156 | -0.321** | -0.337** | -0.215 | -0.467** | -0.337** | -0.025 | -0.105 | -0.247* |
| HeNP |  |  |  |  |  |  | 1 | -0.089 | 0.133 | 0.180 | -0.241* | -0.360** | -0.177 | -0.454* | -0.131 | 0.122 | 0.017 | -0.083 |
| SM |  |  |  |  |  |  |  | 1 | 0.326** | -0.207 | 0.725** | 0.636** | 0.257* | 0.574** | -0.034 | -0.116 | -0.215 | 0.104 |
| pH |  |  |  |  |  |  |  |  | 1 | -0.171 | 0.522** | 0.163 | -0.240* | 0.234* | 0.215 | -0.219 | -0.210 | 0.158 |
| BD |  |  |  |  |  |  |  |  |  | 1 | -0.354** | -0.368* | -0.024 | -0.137 | -0.086 | 0.216 | 0.120 | 0.010 |
| DOC |  |  |  |  |  |  |  |  |  |  | 1 | 0.807** | 0.199 | 0.616** | 0.321** | -0.262* | -0.241* | 0.232 |
| NH4+ |  |  |  |  |  |  |  |  |  |  |  | 1 | 0.295* | 0.535** | 0.192 | -0.213 | -0.251* | 0.125 |
| NO3- |  |  |  |  |  |  |  |  |  |  |  |  | 1 | 0.201 | 0.054 | 0.157 | 0.041 | 0.283* |
| AP |  |  |  |  |  |  |  |  |  |  |  |  |  | 1 | 0.219 | -0.098 | -0.163 | 0.118 |
| SC |  |  |  |  |  |  |  |  |  |  |  |  |  |  | 1 | -0.236* | -0.322** | 0.328** |
| SCS |  |  |  |  |  |  |  |  |  |  |  |  |  |  |  | 1 | 0.704** | 0.472** |
| DBH |  |  |  |  |  |  |  |  |  |  |  |  |  |  |  |  | 1 | 0.464** |
| SH |  |  |  |  |  |  |  |  |  |  |  |  |  |  |  |  |  | 1 |

SOC: soil organic carbon concentration, STN: soil total nitrogen concentration, STP: soil total phosphorus concentration, SCN: soil C:N ratio, SCP: soil C:P ratio, SNP: soil N:P ratio, MBC: soil microbial biomass carbon concentration, MBN: soil microbial biomass nitrogen concentration, MBP: soil microbial biomass phosphorus concentration, SMBCN: soil microbial biomass C:N ratio, SMBCP: soil microbial biomass C:P ratio, SMBNP: soil microbial biomass N:P ratio, LeOC: *Sophora moorcroftiana* leaf organic carbon concentration, LeTN: *Sophora moorcroftiana* leaf total nitrogen concentration, LeTP: *Sophora moorcroftiana* leaf total phosphorus concentration, LeCN: *Sophora moorcroftiana* leaf C:N ratio, LeCP: *Sophora moorcroftiana* leaf C:P ratio, LeNP: *Sophora moorcroftiana* leaf N:P ratio, LiOC: litter organic carbon concentration, LiTN: litter total nitrogen concentration, LiTP: litter total phosphorus concentration, LiCN: litter C:N ratio, LiCP: litter C:P ratio, LiNP: litter N:P ratio, HeOC: understorey herb biomass organic carbon concentration, HeTN: understorey herb biomass total nitrogen concentration, HeTP: understorey herb biomass total phosphorus concentration, HeCN: understorey herb biomass C:N ratio, HeCP: understorey herb biomass C:P ratio, HeNP: understorey herb biomass N:P ratio, SM: soil moisture, pH: soil pH, BD: soil bulk density, DOC: dissolved organic carbon concentration, NH_4_^+^: NH_4_^+^-N, NO_3_^-^: NO_3_^—^N, AP: soil available phosphorus, SC: *Sophora moorcroftiana* coverage, SCS: shrub crown size, DBH: Diameter at breast height of *Sophora moorcroftiana*, SH: *Sophora moorcroftiana* height.
